# Effects of Seawater Salinity and Temperature on Growth and Pigment Contents in *Hypnea cervicornis* J. Agardh (Gigartinales, Rhodophyta)

**DOI:** 10.1155/2013/594308

**Published:** 2013-11-19

**Authors:** Lanping Ding, Yuanyuan Ma, Bingxin Huang, Shanwen Chen

**Affiliations:** Shantou University, Shantou 515063, China

## Abstract

This study simulated outdoor environmental living conditions and observed the growth rates and changes of several photosynthetic pigments (Chl a, Car, PE, and PC) in *Hypnea cervicornis* J. Agardh (Gigartinales, Rhodophyta) by setting up different ranges of salinity (25, 30, 35, 40, 45, and 50) and temperature (15, 20, 25, and 30°C). At conditions of culture, the results are as follows. (1) Changes in salinity and temperature have significant effects on the growth of *H. cervicornis*. The growth rates first increase then decrease as the temperature increases, while growth tends to decline as salinity increases. The optimum salinity and temperature conditions for growth are 25 and 25°C, respectively. (2) Salinity and temperature have significant or extremely significant effects on photosynthetic pigments (Chl a, Car, PE, and PC) in *H. cervicornis*. The results of this study are advantageous to ensure propagation and economic development of this species in the southern sea area of China.

## 1. Introduction

Genus* Hypnea* is the red algae which grows in warm seas [1] on rocks near the intertidal zone. 

Some species have been studied worldwide, such as *Hypnea musciformis* [2–13], *H. boergesenii* [14], *H. cervicornis* [13, 15], *H. charoides* [14, 16–18], *H. cornuta* [19], and *H. japonica* [14, 20], including some new species, such as *H. asiatica* [21], *H. flexicaulis* [16, 22, 23], and *H. valentiae* [24]. In morphology, Abbott [2] described the morphological structure and habit of *H. musciformis *in detail as early as 1999. Wolf et al. [23] identified exotic *H. flexicaulis *in the Mediterranean Sea with a description of morphology and analysis of DNA barcoding *rbcL *and* coxI*. Geraldino et al. [21] identified *H. asiatica* with morphology and nrDNA SSU, plastid *rbcL*, and mitochondrial *cox1*. In marine culture, Ganesan et al. [3] studied the viable cultivation of *H. musciformis* by coir rope on the southeast coast of India. Humm and Krewzer [11] reported the growth rate of *H. musciformis *in the Caribbean Sea. In reproduction, Kong and Ang Jr. [17] and Cecere et al. [19] studied *H. charoides *and *H. cornuta*, respectively. Most reports related to the carrageenan [5, 6, 12, 18]. Some reports also investigated other chemical ingredients such as those of Nagano et al. [13] and Figueiredo et al. [15] who studied lectin from *H. cervicornis*. Hori et al. [20] analysed primary structures of two hemagglutinins from* Hypnea japonica*. Bultel-Poncé et al. [10] isolated and extracted new ketosteroids from *H. musciformis*. In environmental effects, Rathinam et al. [24] reported the biosorption of cadmium metal ion by using *H. valentiae* biomass.

There are few literatures referring to *Hypnea* in China. In morphology, Zhang et al. [25] made a comparative morphological study between *H. musciformis* and* H. japonica*. Xia and Wang [26] described the classification and identification of some *Hypnea* species from China, “Flora algarum marinarum sinicarum (Tomus II Rhodophyta no. IV)” [27] introduced seven kinds of *Hypnea* in detail. In addition, Li [28] characterized the life-history of *Hypnea*. Most researchers focused on the chemical constituents of *Hypnea*. Zhou et al. [1] and Chen et al. [29] reported trace elements and amino acids of *Hypnea* and measuring methods. Zhao et al. [30] and Liu [31] studied the mechanism of extracting high-gelation property carrageenan from *Hypnea*. Some literature also reports on the microstructure and other chemical components of *Hypnea* [32–34]. The studies in China have involved mainly three *Hypnea* species: *H. cervicornis* [1], *H. charoides* [1, 34], and *H. pannosa* [1].

According to investigation, *Hypnea *resources are very abundant in the South China Sea along the coastline of Guangdong, Yangjiang, Shantou, and Hainan provinces [32]. *Hypnea* is an important economic algae which is used for food among people and also as a raw material for size, paint, and agar-agar [31]. At present, its industrial and economical value has not been attached enough importance for development, so there are few reports about the aspects of cultivation biology. *Gelidium* and *Gracilaria* addressed the current lack of raw materials by recommending the expanded use of *Hypnea* as the raw material to make agar-agar in the future [28].

In recent years, though scholars at home and abroad have completed more studies on different aspects of *Hypnea*, there have been no literature reports about the effects of salinity and temperature on the growth process in *H. cervicornis*. The seawater salinity and temperature both are basic environmental factors that have effects on the growth of macroalgae. This paper used *H. cervicornis* as material and explored ranges of salinity and temperature for its optimum growth. Certain theoretical foundations and production guidelines are provided for cultivation and utilization of *H. cervicornis* in the future.

## 2. Materials and Methods

### 2.1. *Hypnea cervicornis* J. Agardh (Gigartinales, Rhodophyta) and Seawater

**Figure 1:**
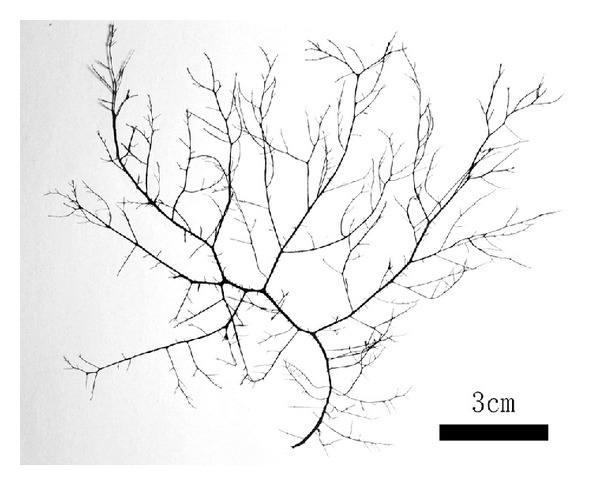
The thallus of *Hypnea cervicornis*.

The sample of *Hypnea cervicornis *was harvested from rocks in the mesolittoral zone at Shenaowan, Nanao Island, Shantou, China (Figure 1). Seawater was taken from Laiwu Ferry and precipitated in a dark environment then filtrated with three layers of filter paper, boiled, sterilized, and cooled before using. *H. cervicornis *used natural seawater to culture for two days at room temperature after taking the sample; then *Hypnea* of similar size with few branches was chosen and its surface scrubbed clean by a brush. Initial seawater salinity was 24 and f1 mother liquid was added to the sterilized seawater as a nutrient solution in the proportion of 1 : 1000 mL. The nutrient solutions were compounded with NaCl and distilled water in order to achieve salinity concentrations of 25, 30, 35, 40, 45, and 50, respectively. The salinity was measured by an ATC hand-held-type salimeter. Illumination was provided by a GXZ-type intelligent illumination incubator (made by Ningbo Jiangnan Instrument Factory) for cultivation which was the artificial light source (fluorescent tube). This incubator was equipped with a digital display regulator that showed the temperature inside of it. The temperatures of cultivation were set at 15, 20, 25, and 30°C.

### 2.2. Cultures and Other Conditions

*Hypnea* was cultured by illumination incubator by first weighing the disposed materials and measuring the initial photosynthetic activity and pigment contents, then, respectively, placing it in a marked culture dish that contained the above compounded nutrient solution (Table 1). The volume of nutrient solution added to the culture dish was based on the materials' weight, which could not exceed 2/3 volume of the dish in order to ensure that supplement and nutrition demand were basically the same. Other conditions: initial weight was about 0.5 g, intensity of illumination was 29.41–39.22 *μ*mol·m^−2^·s^−1^, photoperiod with 12 : 12 h light/dark cycle, and experimental period was 15 d. Medium renewal was carried out every five days. The morphology of *Hypnea* was observed and its weight and photosynthetic activity were measured before renewal in order to document the condition of growth. Notes were taken every 5 days, four times in all (including initial value). Pigment (Chl a, Car, PE, and PC) contents were measured after finishing the culture. Three parallel groups were set up according to the same conditions.

### 2.3. Light Irradiance and Photoperiod

Four light irradiances were measured in the light incubator by a ZDS-10-type automatic shift digital luxmeter (made by Shanghai Jiading Xuelian Meter Factory). The average value obtained was the light irradiance for the experiment. Photoperiod was set by the intelligent illumination incubator.

### 2.4. Weight and Photosynthetic Activity

 The cultured *Hypnea* was removed by tweezers and unnecessary water eliminated by absorbent paper. The *Hypnea* was then placed in a box and weighed on an electronic balance. Photosynthetic activity is an effective assessment of photosynthesis which reflects the light utilization rate of *Hypnea*, thus indicating its condition of growth. The photosynthetic activity of *Hypnea* was measured by chlorophyll fluorescence.

### 2.5. Pigment Contents

Chl a and Car contents were obtained by the following method: 0.1 g (FW) *Hypnea* was weighed after culturing *n* days and extracted by 5 mL 100% carbinol, then placed in a 4°C refrigerator (preserved by avoiding light), stood 24 h, centrifuged at 5000 r for 10 minutes, at 4°C. Absorbance of the extracts was measured by UV-Vis spectrophotometer. The reference solution was carbinol. PE and PC contents were obtained by the following method: 0.1 g *Hypnea* (EW) was ground in a phosphate buffer of 0.1 mol·L^−1^ and pH 6.8, constant volume and centrifuged at 5000 r for 10 minutes. Liquid supernatant was taken to measure the absorbance under wavelengths of 455, 564, 592, 618, and 645.

### 2.6. Counts

Computational formula on growth rate SGR (%/d): SGR = [(*W*_*t*_/*W*_0_)^1/*t*^ − 1] × 100%, among which SGR is specific growth rate, *W*_0_ is green weight of *Hypnea* when experiment began (unit: g), *W*_*t*_ is the green weight at *t* time (unit: g), and *t* is spacing interval between two measurement times (unit: d). Computational formulae for pigment contents

- *ω*(Chl) = 16.29∗A665 − 8.54∗A652∗5/1000/*W*_0_,
- *ω*(Car) = 7.6∗(A480 − 1.49∗A510)∗5/1000/*W*_0_,
- *ω*(PE = [(A564 − A592)−(A455 − A592)∗0.2]∗0.12∗1000∗5/1000/*W*_0_,
- *ω*(PC) = [(A618 − A645)−(A592 − A645)∗0.51]∗0.15∗1000∗5/1000/*W*_0_. All units are mg·g^−1^ FW.

### 2.7. Statistical Analysis

The experimental data were analyzed with Excel 2007 software. Statistical analyses for differences between groups were performed using two-way ANOVA followed by GraphPad Prism 5 Demo, conspicuous levels were set as *P* < 0.05 and *P* < 0.01, and *P* < 0.05 was considered statistically significant. *P < 0.05 and **P < 0.01.

## 3. Results

### 3.1. Effects of Salinity on Growth of *H. cervicornis*

**Figure 2:**
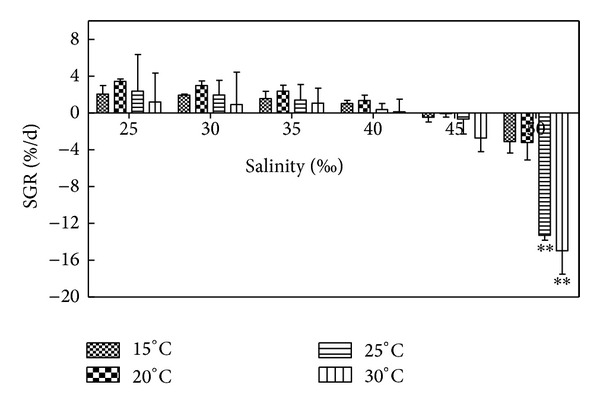
Salinity and temperature effects on SGR (%/d) of *H. cervicornis* cultured for 15 days in sterile seawater enriched with f1 mother liquid and compounded to different salinities, placed in different intelligent illumination incubators with regulated temperature and light intensity. Growth rates were measured once every five days, three times in all; finally the average value was obtained. Treatments with distinct asterisk indicate significant differences according to the two-way ANOVA (GraphPad Prism 5 Demo); **P < 0.01.

The growth of *Hypnea* was slow within a week after beginning the experiment, but *Hypnea* began to branch and became thick with extensions during the culture time (Table 2). As shown in Figure 2, the SGR of *Hypnea* decreased as salinity increased at the same temperature. The algae grew in the 25–40 range of experimental salinity, but the growth effect was better in 25–30. This can be explained by analysis of Figure 2 and Table 2. In suitable salinity, such as 25, the highest weight of *Hypnea* attained was 1.0601 g after 15 days of culture. In unsuitable salinity, such as 50, wet weight of *Hypnea* eventually was reduced to 0.0275 g; high salinity of 45 and 50 showed negative growth, which indicates that salinity conditions are not suitable for the growth of *Hypnea*. It was observed that the color of *Hypnea* was amaranth with many branches in salinity of 25–30; while in other salinity conditions, the color of* Hypnea* lightened, branches were broken easily, and part of which whitened (Table 2). We can see from Figure 2 that two groups at 25°C, 50 and 30°C, 50 showed significant difference (*P* < 0.05) compared to other groups. There were no significant differences (*P* > 0.05) among other groups.

### 3.2. Effects of Temperature on the Growth of *H. cervicornis*

According to Figure 2 and the results of statistical analysis, except for the 50 salinity condition, all temperature groups showed no significant difference (*P* > 0.05). At 15°C, the growth of *Hypnea *was slow. 20–25°C were suitable temperatures for the growth of *Hypnea*. At this temperature, the color of *Hypnea* was amaranth with thick and abundant branches (Table 2), and in suitable temperatures, such as 25°C, the highest weight of *Hypnea* attained was 1.0601 g after 15 days of culture while the highest growth rate attained was 5.37%/d in unsuitable temperature, such as 30°C. The wet weight of *Hypnea* eventually was reduced to 0.0275 g and the growth rate can be as low as −17.04%/d in 30°C. The *Hypnea* grew very quickly in early culture, but the color lightened after culturing for more than 10 days and began to whiten followed by rapid death (Table 2). At 15°C, although the growth of *Hypnea* was slow, the survival time was longer than 25°C and above, and in high salinity of 45 and 50, the death rates of* Hypnea* at 15°C and 20°C were greatly lower than 25°C and above. Figure 2 and Table 2 reflect the growth of *Hypnea* observed under these varied conditions.

#### 3.2.1. Remark

**Figure 3:**
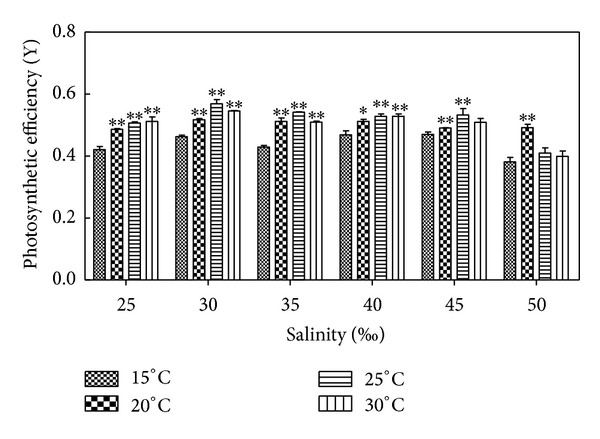
Salinity and temperature effects on photosynthetic activity of *H. cervicornis* cultured for 15 days in sterile seawater enriched with f1 mother liquid and compounded to different salinities, placed in different intelligent illumination incubators with regulated temperature and light intensity. The photosynthetic activities were measured once every five days, four times in all (containing initial value); finally the average value was obtained. Treatments with distinct asterisk indicate significant differences according to the two-way ANOVA (GraphPad Prism 5 Demo); *P < 0.05 and **P < 0.01.

**Figure 4:**
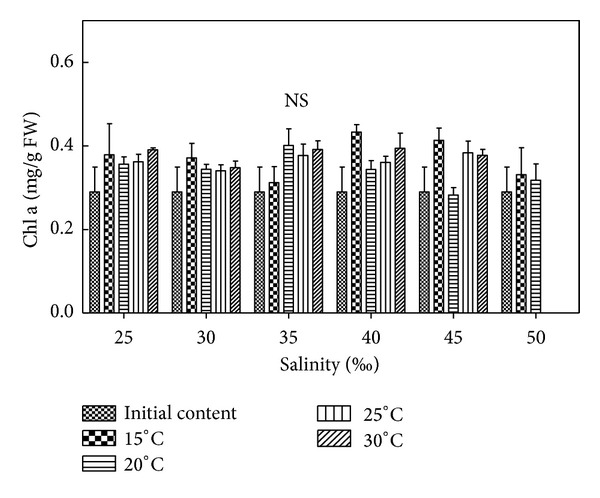
Salinity and temperature effects on Chl a contents of *H. cervicornis* cultured for 15 days in sterile seawater enriched with f1 mother liquid and compounded to different salinities, placed in different intelligent illumination incubators with regulated temperature and light intensity. Treatments with distinct asterisk indicate significant differences according to the two-way ANOVA (GraphPad Prism 5 Demo). NS: nonsignificant (*P* > 0.05).

**Figure 5:**
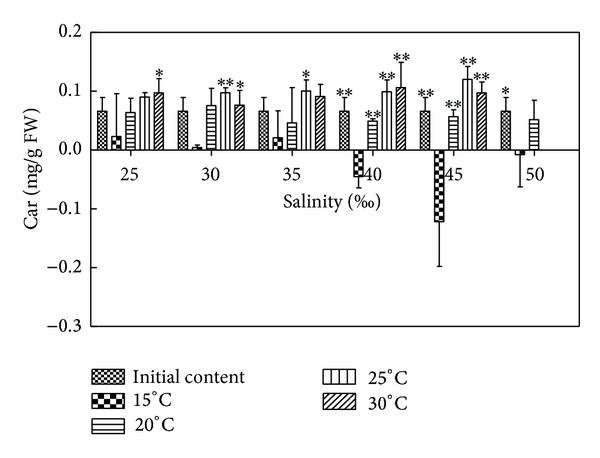
Salinity and temperature effects on Car contents of *H. cervicornis* cultured for 15 days in sterile seawater enriched with f1 mother liquid and compounded to different salinities, placed in different intelligent illumination incubator with regulated temperature and light intensity. Treatments with distinct asterisk indicate significant differences according to the two-way ANOVA (GraphPad Prism 5 Demo); *P < 0.05 and **P < 0.01.

**Figure 6:**
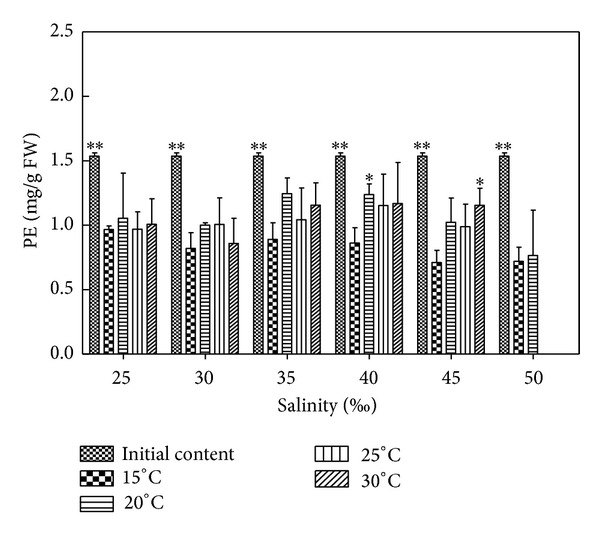
Salinity and temperature effects on PE contents of *H. cervicornis* cultured for 15 days in sterile seawater enriched with f1 mother liquid and compounded to different salinities, placed in different intelligent illumination incubators with regulated temperature and light intensity. Treatments with distinct asterisk indicate significant differences according to the two-way ANOVA (GraphPad Prism 5 Demo); *P < 0.05 and **P < 0.01.

**Figure 7:**
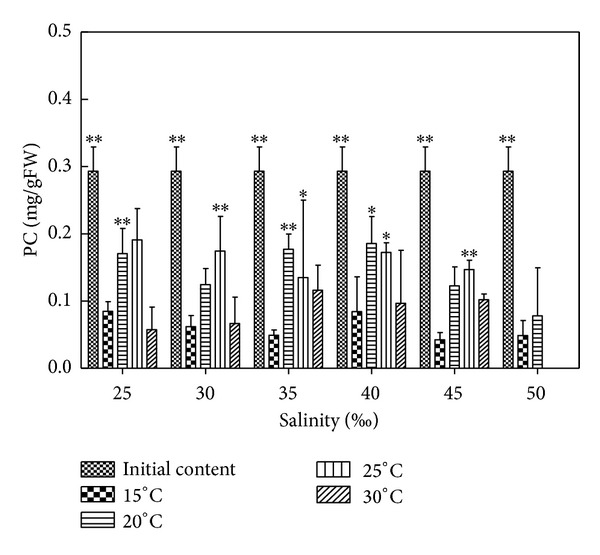
Salinity and temperature effects on PC contents of *H. cervicornis* cultured for 15 days in sterile seawater enriched with f1 mother liquid and compounded to different salinities, placed in different intelligent illumination incubators with regulated temperature and light intensity. Treatments with distinct asterisk indicate significant differences according to the two-way ANOVA (GraphPad Prism 5 Demo); *P < 0.05 and **P < 0.01.

Statistical analysis of Figures 2–7 only showed differences among each salinity set in detail at 15°C compared to the other temperature conditions. The comparison among other temperature conditions can be seen in the description. 

### 3.3. Effects of Salinity on Photosynthetic Activity of *H. cervicornis*

According to Figure 3 and results of statistical analysis, the photosynthetic activity of *Hypnea* did not follow a certain pattern in different salinity. In general, the 25–40 concentration compared to the high salinity concentration of 45 and 50 were significantly different (*P* < 0.05) or showed extremely significant differences (*P* < 0.01). In Figure 3, the activity of *Hypnea *showed no significant difference (*P* > 0.05) in salinity 25–35, which indicated very low death rates compared to very high in high salinity of 45 and 50 indicating that this salinity condition is not suitable indeed for the growth of *Hypnea*. This demonstrates that the range of salinity between 25 and 40 is suitable for the growth of *Hypnea*, and in suitable salinity, such as 30, the minimum of the photosynthetic activity on *Hypnea* dropped to 0.568 Y after 15 days of culture, which decreased slowly (because light intensity was not suitable, the photosynthetic activity has been decreasing, and initial photosynthetic activity was 0.604 Y). In unsuitable salinity, such as 50, the minimum of the photosynthetic activity on *Hypnea* dropped to 0.182 Y, which indicated significant decline. Figure 3 reflects the observed photosynthetic activity of *Hypnea*.

### 3.4. Effects of Temperature on the Photosynthetic Activity of *H. cervicornis*

According to Figure 3 and results of statistical analysis, the photosynthetic activity of *Hypnea* followed a certain pattern at different temperatures. Photosynthetic activity increased first then decreased as temperature increased. Between 20°C and 30°C compared to 15°C, salinity under 40 showed significant difference (*P* < 0.05) or extremely significant difference (*P* < 0.01) in high salinity (45 and 50); 20°C and 25°C compared to other temperatures indicated extremely significant difference (*P* < 0.01); 20°C compared to 25°C showed no significant difference (*P* > 0.05) in 25–40, while the two showed significant difference (*P* < 0.05) or extremely significant difference (*P* < 0.01) in high salinity (45 and 50); 20°C compared to 30°C, except for 50 which showed extremely significant difference (*P* < 0.01). The others indicated no significant difference (*P* > 0.05); there were no significant difference (*P* > 0.05) between 25°C and 30°C. The activity of *Hypnea* was the highest in 20°C except for high salinity of 50; the other salinity had the highest activity of *Hypnea* in 25°C. In range of experimental salinity, the activity of *Hypnea* was the lowest in 15°C, which indicates that 15°C is not suitable for the growth of *Hypnea*. Although the activity of *Hypnea* was high in 30°C, it was observed by the naked eye that if *Hypnea* was cultured for a long time in this temperature, death rates occurred very quickly. This shows that the temperature range of 20–25°C is suitable for the growth of *Hypnea*, and in suitable temperature, such as 25°C, the minimum of the photosynthetic activity in *Hypnea* dropped to 0.568 Y after 15 days of culture, which decreased slowly; however, in unsuitable temperature, such as 30°C, the minimum of the photosynthetic activity in *Hypnea* dropped to 0.182 Y, which was reduced significantly. Figure 3 reflects the observed photosynthetic activity of *Hypnea*.

### 3.5. Effects of Salinity and Temperature on Chl a Content of *H. cervicornis*

According to Figure 4 and results of statistical analysis, there were no significant differences (*P* > 0.05) between each group. Relative to initial content of Chl a, all *Hypnea *died in temperatures of 25°C and 30°C in high salinity 50 and pigment contents cannot be measured. The Chl a contents of *Hypnea* increased in other conditions. In 15°C and 30°C, the Chl a contents of *Hypnea* were high relative to different salinity. The Chl a contents were moderate in 20–25°C, and in salinity 25–35, the Chl a contents of *Hypnea* were high in 20°C and 25°C. In suitable conditions, such as 20°C and 35, the Chl a contents attained 0.4802 mg/g FW, which increased by 13.48% relative to the initial content, increased by 18.77% compared with adjacent temperatures (such as 15°C and 35), and increased by 13.96% compared with adjacent salinity (such as 20°C and 40). In unsuitable conditions, such as 15°C and 50, the Chl a contents were reduced to 0.2231 mg/g FW, which decreased by 12.23% relative to the initial content, decreased by 7.16% compared with adjacent temperatures (such as 20°C and 50), and decreased by 17.7% compared with adjacent salinity conditions (such as 15°C and 45).

### 3.6. Effects of Salinity and Temperature on Car Content of *H. cervicornis*

According to Figure 5 and results of statistical analysis, each salinity group compared to initial content, except for 15°C and 40–50, was of significant difference (*P* < 0.05) or of extremely significant difference (*P* < 0.01). The other groups indicated no significant difference (*P* > 0.05); 15°C compared to 20°C, salinity 25–35 and 50 showed no significant difference (*P* > 0.05), salinity 40–45 indicated extremely significant difference (*P* < 0.01); 15°C compared to 25°C, except for 25 showed no significant difference (*P* > 0.05). The other salinity conditions showed significant difference (*P* < 0.05) or extremely significant difference (*P* < 0.01); the comparisons between 20°C, 25°C, and 30°C showed no significant difference (*P* > 0.05). In different salinity conditions, the Car contents of *Hypnea* decreased relative to the initial content in 15°C, while Car contents increased in 25–30°C. The Car contents of *Hypnea* increased in 20°C only in 25–30. In suitable conditions, such as 25°C and 35, the Car contents attained 0.1178 mg/g FW, which increased by 3.83% relative to the initial content, increased by 3.30% compared with adjacent temperatures (such as 30°C and 35), and increased by 1.71% compared with adjacent salinity (such as 25°C and 40). In unsuitable conditions, such as 15°C and 45, the Car contents were reduced to −0.2068 mg/g FW, which decreased by 28.63% relative to the initial content, decreased by 26.69% compared with adjacent temperatures (such as 20°C and 40), and decreased by 15.5% compared with adjacent salinity (such as 15°C and 40).

### 3.7. Effects of Salinity and Temperature on PE Content of *H. cervicornis*

According to Figure 6 and results of statistical analysis, each temperature group compared to initial content indicated no significant difference (*P* > 0.05) in 15°C, except for 35–40 showed no significant difference (*P* > 0.05) and the other conditions showed extremely significant difference (*P* < 0.01) in 20°C. Each salinity condition showed significant difference (*P* < 0.05) or extremely significant difference (*P* < 0.01) in 25°C; except for 40 which showed no significant difference (*P* > 0.05) and the other conditions which indicated significant difference (*P* < 0.05) or extremely significant difference (*P* < 0.01) in 20°C; 15°C compared to 20°C, except for 40 indicated significant difference (*P* < 0.05) and the other conditions showed no significant difference (*P* > 0.05); 15°C compared to 25°C, indicated no significant difference (*P* > 0.05); 15°C compared to 30°C, except for 45, showed significant difference (*P* < 0.05) and the other conditions showed no significant difference (*P* > 0.05). The comparisons between 20°C, 25°C, and 30°C indicated no significant difference (*P* > 0.05). Relative to the initial content of PE, the PE contents of *Hypnea* decreased in different salinities and temperatures. It was observed by the naked eyes that the color of *Hypnea *gradually turned brown with prolonged culture time. The PE contents of *Hypnea* were lower than the other temperature groups in 15°C. Relative to the other salinity groups, the PE contents of *Hypnea* were high in salinity 35–40. In suitable conditions, such as 20°C and 25, the PE contents can be reduced to 1.4272 mg/g FW, which decreased by 8.15% relative to the initial content, decreased by 47.64% less than adjacent temperature (such as 15°C and 25), and decreased by 41.84% less than adjacent salinity (such as 20°C and 30). In unsuitable conditions, such as 15°C and 50, the PE contents were reduced to 0.5934 mg/g FW, which decreased by 91.53% relative to the initial content, decreased by 8.86% compared with adjacent temperature (such as 20°C and 50), and decreased by 11.47% compared with adjacent salinity (such as 15°C and 45).

### 3.8. Effects of Salinity and Temperature on PC Content of *H. cervicornis*

According to Figure 7 and results of statistical analysis, each temperature group compared to initial content indicated significant difference (*P* < 0.05) or extremely significant difference (*P* < 0.01); 15°C compared to 20°C, except for salinity 35–40, showed significant difference (*P* < 0.05) or extremely significant difference (*P* < 0.01) and the other conditions indicated no significant difference (*P* > 0.05); 15°C compared to 25°C showed significant difference (*P* < 0.05) or extremely significant difference (*P* < 0.01); 15°C compared to 30°C indicated no significant difference (*P* > 0.05); 20°C compared to 25°C showed no significant difference (*P* > 0.05); 20°C compared to 30°C, except for salinity 25 and 40, showed significant difference (*P* < 0.05) or extremely significant difference (*P* < 0.01) and the other salinity conditions showed no significant difference (*P* > 0.05); 25°C compared to 30°C, except for salinity 25–30, showed extremely significant difference (*P* < 0.01) and the other salinity conditions indicated no significant difference (*P* > 0.05). The PC contents of *Hypnea* decreased in different salinity and temperature, which were very low in 15°C and 30°C; the PC contents of *Hypnea* in 20°C and 25°C were larger than in 15°C and 30°C; relative to high salinity groups (45 and 50), the PC contents of *Hypnea *were high in salinity 25–40. In suitable conditions, such as 25°C and 35, the PC contents were reduced to 0.2662 mg/g FW, which decreased by 1.51% relative to the initial content, decreased by 16.42% less than adjacent temperature (such as 30°C and 35), and decreased by 9.36% less than adjacent salinity (such as 25°C and 40). In unsuitable conditions, such as 15°C and 50, the PC contents were reduced to 0.0291 mg/g FW, which decreased by 25.22% relative to the initial content, decreased by 1.51% compared with adjacent temperature (such as 20°C and 50), and decreased by 1.03% compared with adjacent salinity (such as 15°C and 45).

 Synthesized changes of pigment contents were observed. The Chl a contents were high, but the contents of Car, PE, and PC were low in 15°C. The results of statistical analysis indicated that the Chl a contents showed no significant difference (*P* > 0.05); the Car contents compared to the initial content showed no significant difference (*P* > 0.05) in salinity 25–35, and compared to 20°C, there was no significant difference (*P* > 0.05) in salinity 25–35 and 50, and compared to 25°C, there was no significant difference (*P* > 0.05) in salinity 25, and compared to 30°C, there was no significant difference (*P* > 0.05) in salinity 35. The other conditions indicated a significant difference (*P* < 0.05) or an extremely significant difference (*P* < 0.01). The PE contents in 15°C compared to 20°C showed no significant difference (*P* > 0.05) in salinity 25–35 and 45–50, and compared to 25°C, there was no significant difference (*P* > 0.05), and compared to 30°C, there was no significant difference (*P* > 0.05) in salinity 25–40. The other conditions indicated a significant difference (*P* < 0.05) or an extremely significant difference (*P* < 0.01). The PC contents in 15°C compared to 20°C showed no significant difference (*P* > 0.05) in salinity 25–30 and 45–50, and compared to 30°C, there was no significant difference (*P* > 0.05). The other conditions indicated a significant difference (*P* < 0.05) or an extremely significant difference (*P* < 0.01). The contents of Chl a, Car, PE, and PC were high in 20–25°C (relative to the other conditions). The results of statistical analysis indicated that the contents of Chl a, Car, PE, and PC showed no significant difference (*P* > 0.05) between 20°C and 25°C, and compared to the initial content, the Car contents showed no significant difference (*P* > 0.05). The PE contents except for 20°C and salinity 35–40 showed no significant difference (*P* > 0.05). The other conditions indicated a significant difference (*P* < 0.05) or an extremely significant difference (*P* < 0.01). The PC contents showed a significant difference (*P* < 0.05) or an extremely significant difference (*P* < 0.01). The contents of Chl a, Car, and PE were high in 30°C, but the PC contents were low. The results of statistical analysis indicated that the Chl a contents showed no significant difference (*P* > 0.05); the Car contents compared to the initial content and the contents in 20°C and 25°C showed no significant difference (*P* > 0.05); the PE contents compared to the initial content, except for salinity 40, showed no significant difference (*P* > 0.05). The other conditions indicated a significant difference (*P* < 0.05) or an extremely significant difference (*P* < 0.01), and compared to 20°C and 25°C, there was no significant difference (*P* > 0.05); the PC contents compared to the initial content showed an extremely significant difference (*P* < 0.01), and compared to 20°C, except for salinity 25 and 40, showed significant difference (*P* < 0.05) or extremely significant difference (*P* < 0.01). The other conditions indicated no significant difference (*P* > 0.05), and compared to 25°C, except for salinity 25–30, showed an extremely significant difference (*P* < 0.01). The other conditions indicated no significant difference (*P* > 0.05). The PE and PC contents decreased in all temperature groups relative to the initial content of *Hypnea*, the Chl a contents increased, and the Car contents of all salinity groups increased in 25°C and 30°C, while the Car contents increased only in salinity 25 and 30 in 20°C. In summary, this study indicates that the salinity groups 25–30 and the temperature groups 20–25°C are most suitable for the growth of *Hypnea*.

## 4. Discussion

 The results of this study show that the growth of *H. cervicornis *closely relates to the changes in external environmental factors (seawater salinity and temperature) and internal physiological factors (pigment). The growth of *H. cervicornis* under good conditions in temperatures of 25°C and 30°C within 10 days of culture, but wet weight, begins to decrease if cultured for more than 10 days, with rapid decrease especially in 30°C. Although the growth is slow in 15°C, the death rate is low, so it is speculated that low temperature is suitable for preservation. The death rates of *H. cervicornis *in 15°C and 20°C in high salinity (45 and 50) are also much less than in temperatures of 25°C and above; therefore, if the culture time is not long, *H. cervicornis* can adapt to a temperature of about 25°C. If the culture time is long, it can adapt to a temperature of about 20°C, and the seawater salinity of cultivation should not exceed salinity 40. It agrees with the temperature conditions of *H. cervicornis *grown for 3-4 months. The natural seawater salinity in the Guangdong area is also in the range of 24–30. It is demonstrated that *H. cervicornis *can adapt in the long term to the environment and a scientific basis is provided for the further regulation of the growth and development of it.

 Temperature is an important environmental factor that affects the growth and development of *H. cervicornis*. The temperature range is different for different species and different processes of growth and development. For example, Guist et al. [35] found that the biomass of *H. musciformis* increased by 20% every day and the growth rate was the highest when the water temperature was 18–24°C, which was inversely proportional to the biomass and to the level of solar irradiance. Pan [36] found that the color of *Porphyra haitanensis *free conchocelis was yellow and showed poor growth if the temperature was above 23°C, and when above 29°C, the growth of the free conchocelis would stop, phycoerythrin disappeared and became yellowish-brown; when the temperature increased above 30°C, the free conchocelis showed poor growth and even displayed the death phenomenon. Liu and Dong [37] found that light and temperature have an important influence on the growth and biochemical component of *Gracilaria tenuistipitata*. The influence of these two factors had an interaction function: growth rate of *G. tenuistipitata* increased as temperature increased in range of 15–25°C and decreased in 30°C. *Hypnea *is an autotrophic plant and cannot live without photosynthesis in any conditions. Physiological processes are closely related to temperature and this study indicates that temperature can influence the growth and photosynthetic activity of *H. cervicornis*, which grows in the range of 15–25°C. The growth rate begins to decrease when cultured above 10 days in 30°C, part of *Hypnea* whitens and dies.

Seawater salinity is also closely related to the growth and photosynthetic activity of *H. cervicornis*. Salinity is a measure of a variety of inorganic salt concentrations in seawater. The salinity level determines the seawater osmotic pressure. For marine algae, the osmotic pressure affects moisture distribution inside and outside of the semipermeable membrane and seaweed absorption of nutrients. The salinity range is also different for different species with different processeses of growth and development. For example, Wang et al. [38] found that the photosynthesis rate decreased when *Gracilaria verrucosa* was moved from high-salinity seawater to low-salinity seawater. Xu et al. [39] found that salinity had a significant effect on the nitrogen absorption function of* Gracilaria tenuistipitata var. liui* and had a maximum absorption rate of N in 20%. Cai [40] found that high- and low-salinity stress significantly inhibited the growth of *Gracilaria lemaneiformis* and hole-shaped contact between cells was damaged in low-salinity (10) stress. Algae chromatoplast structure was damaged to a certain extent in high-salinity (35) stress and caused massive accumulation of salt particles in the cell. The results of this study indicate that in suitable growth temperature *H. cervicornis *can grow in range of salinity 25–40, but the growth rate constantly decreases above salinity 40. If salinity is too high, photosynthetic activity of *H. cervicornis *will be affected and further influence its growth. If the salinity is too low, the photosynthetic activity of *H. cervicornis* is not high.

In preliminary experiments, the activity of *H. cervicornis* decreased at the beginning of cultivation due to instability. The photosynthetic activity of *H. cervicornis *recovered and was above initial activity after a period of cultivation, but it decreased if cultivation continued too long. It was presumed that this was a process of *H. cervicornis* adaptation to its environment. In this experiment where the activity of *H. cervicornis* decreased constantly, we investigated and found that the light intensity of the preliminary experiment was set stronger than this experiment and it is presumed that light has a significant influence on the growth of *H. cervicornis*. Further study is indicated in this area.

Chl a is basic photosynthetic pigment of all organisms that carry out photosynthesis by releasing oxygen and exists in all algae and all photosynthetic organisms except for photosynthetic bacterium. The number of main light-harvesting pigments (Chl a and Car) in *Hypnea* determines the ability to absorb light and further affects the growth rate of the algae. Chl a is an important photosynthetic pigment of *Hypnea*; high or low content is closely related to whether or not vegetative growth of algae itself is vigorous. Phycobiliprotein is one of parameters used to evaluate *Hypnea* quality, which mainly has functions with the following aspects. (1) In growth stage of *Hypnea*, phycobiliprotein is an important accessory pigment in photosynthesis, which affects the photosynthesis efficiency of the algae. (2) It affects algae quality. (3) It has a very high nutritional value and application prospect. Phycobiliprotein is a light-harvesting pigment protein that largely emerges in Rhodophyta, Cyanophyta, and Cryptophyta, which mainly include three types: phycoerythrin, phycocyanin, and allophycocyanin. In phycobilisome, the direction of energy transfer is phycoerythrin-phycocyanin-allophycocyanin, eventually transfers to reaction center pigment Chl a in high-authority, and is used in the photosynthesis. Seen from outside, phycoerythrin is red. The color of phycocyanin changes between pink and navy blue, and allophycocyanin is blue with a little green. It is generally thought that in Cyanophyta and Rhodophyta, the phycocyanin acts as a light-harvesting pigment system to carry out the function of absorption and energy transfer in the photosynthesis in addition to acting as a storage protein in the cell. The external environmental factors of light, temperature, and salinity have important effects on the biochemical components of *Hypnea*, while changes of biochemical components affect color and photosynthetic efficiency of *Hypnea*, and further affect its growth. It indicates that the internal physiological factors such as chlorophyll and phycobilin also have important effects on the growth of *Hypnea*. Lapointe [41] found that light may often limit growth of *Gracilaria foliifera var angustissima* in nature; Chl a and PE contents were inversely proportional to light level and growth rate, but pigment content did not affect the growth capacity of *Gracilaria*. Liu and Dong [37] found that phycoerythrin and chlorophyll contents of *Gracilaria tenuistipitata* decreased if light increased, which increased when the temperature increased. Jin et al. [42] found that the chlorophyll content of *Gracilaria chouae* was at the highest peak in temperature of 17°C, and phycobiliprotein content was the minimum. The chlorophyll and phycobiliprotein contents of *G. chouae *were at the maximum in 30 salinity. The results of this experiment show that Chl a, Car, and phycobiliprotein contents of *H. cervicornis *are high in a salinity range of 25–30 and temperature range 20–25°C relative to other salinity and temperature conditions. If salinity or temperature is too low or too high, the color and growth condition of *H. cervicornis *will be greatly impacted. The fact that the biochemical components of *Hypnea* as shown above change with variations in salinity and temperature is a positive physiological adaptability to nature that has important ecological significance. It also indicates that the growth of *Hypnea* is affected by external environmental factors and internal physiological factors. Salinity, temperature, and the biochemical components of algae are closely related to the growth of *H. cervicornis*.

If *H. cervicornis *was cultured a long time under experimental conditions that produced the negative growth phenomenon, the following reasons may be responsible. (1) Because the volume of culture water was small, it cannot be compared to natural field conditions. *H. cervicorni *was cultured in a culture dish then placed in an incubator for this experiment. The density, volume of water, and the nutrition had great differences from the field environment. Besides, the natural field condition changes every day (such as climate, water salinity, and nutrition), while algae cultured in the room usually has certain fixed conditions. (2) The problem with a vegetative growth period is that algae grows to a certain extent influenced by factors such as the nutrition used up or the algae cells all having matured or unsuitable environmental conditions leading to cells which cannot be updated; then the growth of algae will stop or even die. (3) There is a difference in light intensity and water flow. The light intensity of the field environment is changing constantly because of climate and the amount of sunshine, which varies from strong to weak, while the light intensity in the room is generally fixed in a small range. If not suitable for the growth of* Hypnea*, the growth conditions of algae would necessarily be affected and could not be recovered as the steady light intensity. In addition, seawater in the field environment is constantly flowing and supplying nutrition for the *Hypnea*; thus, the algae cells are updated constantly, and dead organisms of the algae are washed out by flowing seawater. This has a significant advantage in supplying oxygen and has a great influence on the photosynthesis of *Hypnea*. In a word, the growth conditions of *Hypnea* are difficult to keep consistent between lab and field cultivation. If conditions are unsuitable, the *Hypnea *cultivation will show the above-mentioned negative growth phenomenon, and the algae will soften if grown in an unsuitable environment for a long time. The color will turn from amaranth to celadon and the algae will further whiten and die.

## Figures and Tables

**Table 1:** The formula of f1 mother liquid.

Chemical reagent	NaNO_3_	NaH_2_PO_4_	FeC_6_H_5_O_7_	Tris	KI	H_3_BO_3_	VB_1_	VB_12_
Amount (g)	21.249	3.599	0.129	30.283	0.416	15.449	0.126	0.249×10^−3^

The chemical reagents were stirred, respectively, to full dissolution in distilled water and mixture. The solution was diluted to a final volume of 500 mL with distilled water. All reagents were successfully dissolved in solution.

**Table 2:** The morphological changes of *H. cervicornis* in process of cultivation.

Conditions of culture*∖*days of culture	0 d	5 d	10 d	15 d
15°C, 25		Thin thalli, light purple, with few branches	Thalli thickening, color further lightened, with a few of branches increase	Thalli thickened, pale purple, with branches increase
15°C, 30				
15°C, 35				
15°C, 40				
15°C, 45				
15°C, 50		Thalli softened with some whitened and died	A part of thalli whitened and died	Most part of thalli whitened and died
20°C, 25		Thin thalli, light purple, with few branches	Thalli thickened gradually, light purple, with a few of branches increase	Thalli thickened, pale purple, with branches increasing significantly
20°C, 30				
20°C, 35				Thalli thickened, pale purple, with branches increase
20°C, 40				
20°C, 45				Thalli thickened, pale purple, with a few of branches increase
20°C, 50	Thin thalli, purple, with few branches	Thalli softened with some whitened and died	Most part of thalli whitened and died	Most part of thalli died
25°C, 25		Growth of thalli in good condition and thickening, purple, branches start increasing	Thalli thickened, light purple, with branches increasing significantly	Thalli thickened, pale purple with slight green, many branches with a few died
25°C, 30				
25°C, 35			Thalli thickened, light purple, with branches increase	Thalli thickened, pale purple with slight green, many branches with a part died
25°C, 40		Few of thalli whitened and died	Thalli thickened, light purple, with branches increase, few died	Thalli thickened, pale purple with slight green, with many branches, most part of thalli died
25°C, 45		Thalli died significantly	Part of thalli died	Most part of thalli died
25°C, 50			Most part of thalli died	All thalli died
30°C, 25		Growth of thalli in good condition and thickening, purple, with branches increase	Thalli thickened, light purple, with branches increasing significantly, a few died	Thalli thickened, light purple with slight green, with many branches, a part died
30°C, 30				
30°C, 35				
30°C, 40		Few of thalli whitened and died	Thalli thickened, light purple, with branches increase, a few died	Thalli thickened, pale purple with slight green, with many branches, most part died
30°C, 45		Thalli died significantly	Most part of thalli died	Most part of thalli died
30°C, 50			Most part of thalli died	All thalli died
